# Biologically inspired ultrathin arrayed camera for high-contrast and high-resolution imaging

**DOI:** 10.1038/s41377-020-0261-8

**Published:** 2020-02-27

**Authors:** Kisoo Kim, Kyung-Won Jang, Jae-Kwan Ryu, Ki-Hun Jeong

**Affiliations:** 1 Department of Bio and Brain Engineering, Korea Advanced Institute of Science and Technology (KAIST), 291 Daehak-ro, Yuseong-gu, Daejeon, 34141 Republic of Korea; 2 KAIST Institute for Health Science and Technology, KAIST, Daejeon, 34141 Republic of Korea; 3 Unmanned/Robotic Systems Lab., LIG Nex1 Co. Ltd, Seongnam, 13488 Republic of Korea

**Keywords:** Imaging and sensing, Optoelectronic devices and components

## Abstract

Compound eyes found in insects provide intriguing sources of biological inspiration for miniaturised imaging systems. Here, we report an ultrathin arrayed camera inspired by insect eye structures for high-contrast and super-resolution imaging. The ultrathin camera features micro-optical elements (MOEs), i.e., inverted microlenses, multilayered pinhole arrays, and gap spacers on an image sensor. The MOE was fabricated by using repeated photolithography and thermal reflow. The fully packaged camera shows a total track length of 740 μm and a field-of-view (FOV) of 73°. The experimental results demonstrate that the multilayered pinhole of the MOE allows high-contrast imaging by eliminating the optical crosstalk between microlenses. The integral image reconstructed from array images clearly increases the modulation transfer function (MTF) by ~1.57 times compared to that of a single channel image in the ultrathin camera. This ultrathin arrayed camera provides a novel and practical direction for diverse mobile, surveillance or medical applications.

## Introduction

The unique structures of biological vision systems offer intriguing inspiration for ultracompact camera applications^1–3^. Natural insects acquire sufficient visual information with small visual organs of tiny facet lenses^4,5^. Furthermore, compound eyes have superior visual functions, such as large depth-of-field (DOF), wide field-of-view (FOV), high motion sensitivity, and low aberration^6,7^. In particular, the eye structures of an adult *Xenos peckii*, exhibiting hundreds of photoreceptors on an individual eyelet, offer engineering inspiration for ultrathin camera or imaging applications because they have higher visual acuity than other compound eyes^8–10^. For instance, *Xenos peckii*’s eye-inspired cameras provide an ~50 times higher spatial resolution than those from arthropod eyes, i.e., compound eyes with ommatidia^1,10,11^. In addition, the effective image resolution of the *Xenos peckii*’s eye can also be further improved by the image overlap between neighbouring eyelets^8,10^.

Unlike conventional camera lenses, microlenses comparable to insect facet lenses have a relatively small focal length as well as low aberration^12,13^, which can substantially reduce the total track length, i.e., the distance from an image sensor to a lens top, of a camera^14–16^. In addition, these lenses can provide a large DOF due to a small focal length and a small aperture diameter, which allows near-to-infinity imaging^17^. Recently, diverse microfabrication methods of microlens arrays, such as thermal reflow, inkjet printing or 3D direct laser writing, have been actively incorporated with biologically inspired cameras^15,18,19^. However, these methods are still under development to prevent optical crosstalk between microlenses for high-contrast imaging.

Natural compound eyes often have pigment cells for either blocking the ambient optical noise between facet lenses or regulating the amount of incoming light for the external light environment^20–22^. In particular, *Xenos peckii*’s eyes contain pigmented cups surrounding each eyelet to block the incoming off-axis light^8^. Light absorbers such as pigment cells serve as a crucial optical element for high-contrast and high-resolution cameras by reducing the optical crosstalk between microlenses^10,23^. However, conventional light absorbers such as glass stacked diaphragm arrays or machined baffle arrays still have some technical limitations in decreasing the total track length (TTL) of the camera^24,25^. Recently, the replica moulding of black silicone or silicon nanowires also provides optical isolation between microlenses; however, they have some structural restrictions in controlling the amount of incoming light through microlenses^11,26^. In addition, the complicated integration of optical elements still has some restrictions for either aligning the optical axis or improving the image resolution^27^.

**Fig. 1:**
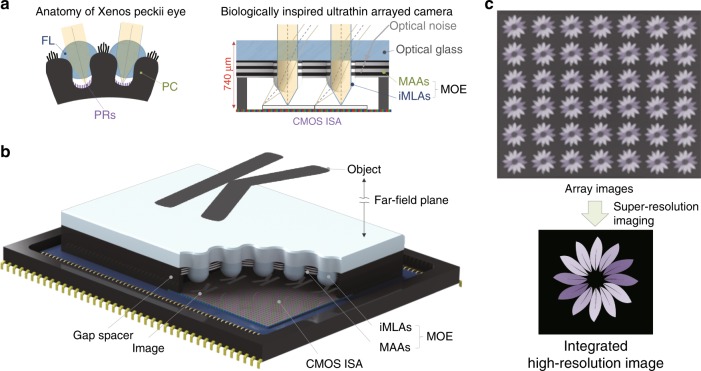
Schematic illustrations of a biologically inspired ultrathin arrayed camera and an image reconstruction method. **a** Biological inspiration: *Xenos peckii*’s eye versus the ultrathin camera. Like the natural eye comprising facet lenses (FL), pigmented cups (PC), and photoreceptors (PRs), the biologically inspired ultrathin camera features inverted microlens arrays (iMLAs), multilayered aperture arrays (MAAs), gap spacers, and CMOS image sensor arrays (CMOS ISA). Both the pigment cups and the MAAs efficiently reduce the optical crosstalk between their lenses and thus increase the image contrast. **b** Imaging concept of the ultrathin arrayed camera. Objects located in the far-field plane are similarly imaged on each channel due to the small visual disparity of each channel. **c** Super-resolution imaging for acquiring high-contrast and high-resolution images from array images

Here, we report an ultrathin arrayed camera for high-contrast and high-resolution imaging, inspired by the vision system of *Xenos peckii* (Fig. 1a). The ultrathin camera consists of multilayered aperture arrays (MAAs), inverted microlens arrays (iMLAs), and gap spacers on a planar CMOS image sensor. The MAAs, stacking UV patterned black polymer circular patterns, serve as cylindrical pinhole arrays, which provide efficient light absorption over the whole visible spectrum and thus substantially reduce the optical crosstalk between microlenses. The iMLAs offer a relatively higher FOV than upward MLAs because the refracted light from a front glass window enters individual microlenses with an additional angle. The FOV of a single channel in the ultrathin camera is determined by the diameter and thickness of the MAAs as well as the focal length of the microlens (Supplementary Fig. S1). For instance, the designed thickness and aperture diameter of MAAs for the 70° FOV are 60 μm and 35 μm, respectively. The image overlap between neighbouring channels can also be precisely controlled by the period of the microlens. The array images from multiple channels are uniform but are slightly different when an object target is located in a far-field plane (Fig. 1b). A single high-resolution image can be reconstructed from the array images by using a multi-frame super-resolution algorithm (Fig. 1c).

## Results and discussion

**Fig. 2:**
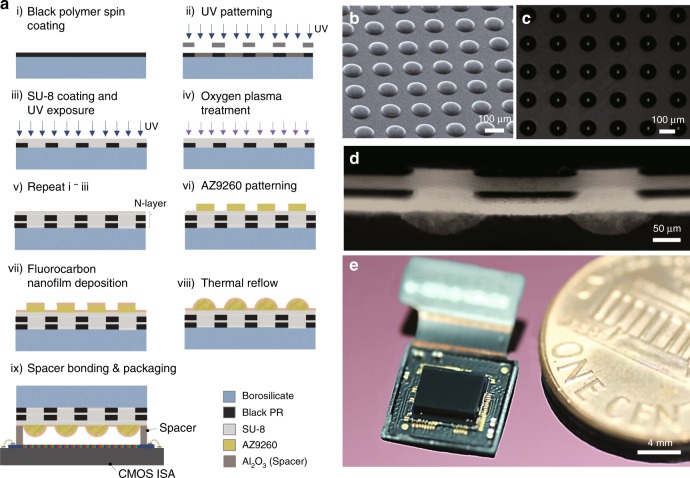
Microfabrication steps and captured images of the MOE, i.e., MAAs and iMLAs, and the ultrathin camera. **a** Microfabrication methods of the MOE, formed by using repeated photolithography and thermal reflow. The MOE was further integrated on a CMOS image sensor with gap spacers by using a flip-chip bonder. **b** Scanning electron microscopy and **c** optical microscopy images of microlens arrays. The white dots on the captured optical image display focused beams through the iMLAs. **d** A cross-sectional optical image of the MOE. **e** A captured photograph of a fully packaged ultrathin arrayed camera

The ultrathin arrayed camera involves the microfabrication of micro-optical elements (MOEs) and the camera packaging (Fig. 2a). For the MOE, a 5-μm thick black photoresist resin (GMC 1040, Gersteltec, Switzerland) was photolithographically defined on a 4-inch borosilicate glass wafer. A 25-μm-thick transparent photoresist resin (SU-8 2025, MicroChem Corp.) was spin-coated on the black layer. Both steps were repeated to construct multiple layers. Hydrophilic treatment using an oxygen plasma was performed after SU-8 patterning to increase the adhesion between the black resin and SU-8. The iMLAs were further formed on the multiple layers by using photolithographic patterning (AZ9260, MicroChem Corp.), a C_4_F_8_-based hydrophobic coating, and thermal reflow. The MOE was diced to 5.1 mm × 5 mm to cover the whole image sensor chip, including an optical black area. Four alumina spacers of 150 μm in thickness were precisely and permanently placed on the CMOS image sensor arrays (CMOS ISA, Sony IMX 219, 8 M pixels, unit pixel: 1.12 μm × 1.12 μm, frame rate: 30 fps) microdispensing an epoxy adhesive. The MOE was then packaged on the CMOS ISA with spacers by using a flip-chip mounter. The fully packaged camera was finally assembled with a Raspberry Pi board. Scanning electron microscopy (SEM) and optical microscopy images show the microfabricated iMLAs (Fig. 2b, c). The captured cross-sectional image of the MOE clearly demonstrates that the MAAs have been successfully fabricated by using repeated photolithography (Fig. 2d). Figure 2e also shows the fully packaged ultrathin arrayed camera with the MOE, where the f-number of the microlens is 1.7, the FOV is 73° and the TTL including a window glass is 740 μm (Supplementary Fig. S2).

**Fig. 3:**
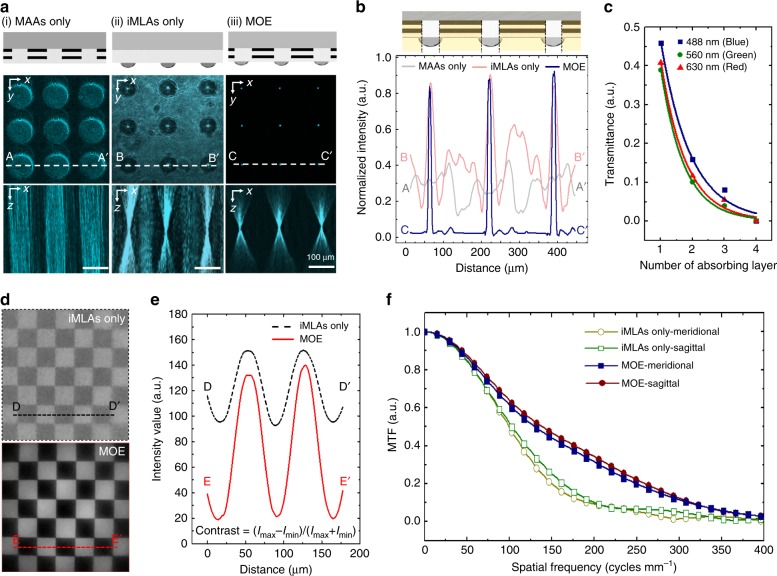
High-contrast imaging through the MOE. **a** Sectioned images of a 532-nm laser beam passing through (i) the MAAs only, (ii) the iMLAs only, and (iii) the MOE measured with a confocal laser scanning microscope. **b** The corresponding normalised intensity profiles along lines AA′, BB′, CC′ on the sectioned optical images, which clearly demonstrate beam focusing and light blocking. **c** Normalised transmittance depending on the number of black polymeric layers at three different wavelengths. Each absorbing layer of a 5-μm-thick black and a 25-μm-thick transparent polymer resin was repeatedly stacked. **d** Camera images captured through the iMLAs only (upper) or with the MOE (lower). **e** The corresponding intensity profiles along lines DD′ and EE′, respectively. The image contrast for the ultrathin arrayed camera with the MAAs exhibits substantial enhancement compared to that for the camera without MAAs. **f** MTF curves calculated along the meridional and sagittal directions of the captured image. The MTF50 for the ultrathin camera with the MOE is 1.37× higher than that for the camera without iMLAs only

High-contrast imaging is successfully achieved by using an ultrathin arrayed camera with the MOE (Fig. 3). A confocal laser scanning microscope (CLSM) offers optically sectioned images through the MOE via a collimated 532 laser beam (Fig. 3a). Unlike the iMLAs only, the experimental results clearly demonstrate that a laser beam is focused through the MOE without any optical crosstalk between microlenses. The intensity profile for the MOE also shows a sharp peak signal without optical crosstalk or noise (Fig. 3b). The normalised transmittance is also measured to evaluate the light absorbance of the MAAs in the visible region depending on the number of absorption layers. Note that each layer consists of 5-μm-thick black resin and 25-μm-thick transparent resin (Fig. 3c). The measured transmittance is ~0.4 for a single absorption layer, which exponentially decays as the number of layers increases. All of the visible light is completely blocked by four layers, and 90% of the light is also reduced by two layers. In addition, the captured images of a checkerboard target clearly indicate that the MOE provides higher contrast than only the iMLAs (Fig. 3d). The calculated Michelson contrast of a single image is increased by 3.21, i.e., 0.77 for the MOE and 0.24 for the iMLAs only. The measured modulation transfer function (MTF) curves also compare the image sharpness along both the sagittal and meridional planes (Fig. 3f). The MTF50, i.e., the spatial frequency at half maximum, of the iMLAs is only 102 cycles mm^−1^ along the sagittal plane, whereas that of the MOE is 140 cycles mm^−1^. As a result, the MTF50 is clearly increased by over 30% for both planes. The pinhole diameter of the MAAs also effectively controls the amount of incoming light onto the iMLAs (Supplementary Fig. S3). A high-contrast image without the glare phenomenon was acquired by adjusting the diameter ratio of the lens and pinhole according to the illumination intensity.

**Fig. 4:**
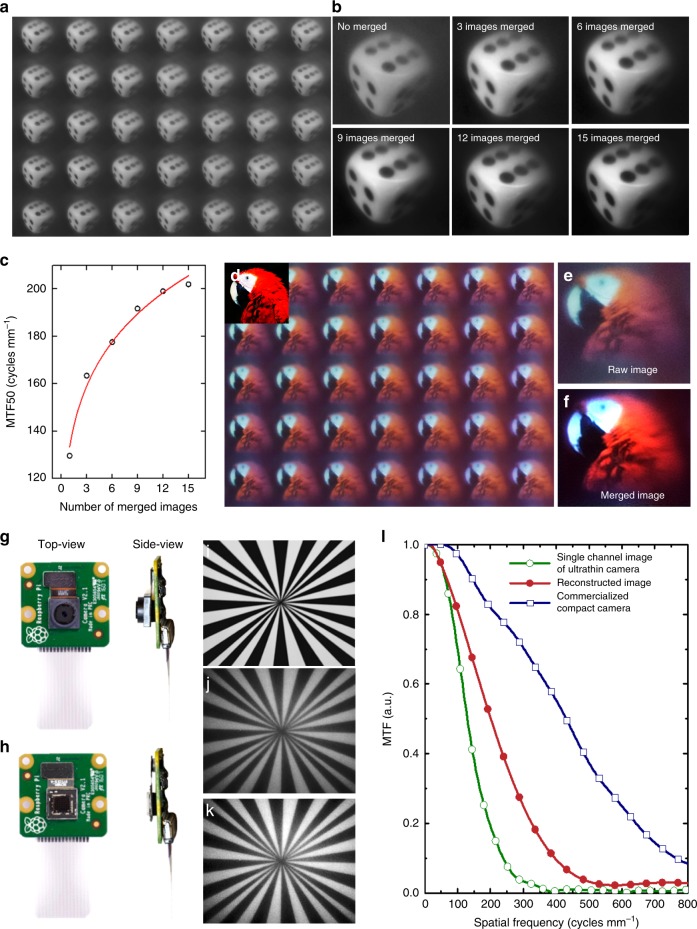
Super-resolution imaging by array images. **a** “Dice” array images captured by the ultrathin arrayed camera. The “dice” test image was displayed on an LED panel at the far-field plane to observe the same figure in each channel. **b** Image reconstructions according to the number of array images by super-resolution imaging. **c** MTF50 graph for the number of merged images. The calculated results show that the combination of array images enhances the image resolution. **d** The captured array images of a red parrot to measure the colour differences between a target object and acquired images. **e** An image captured by a single channel of the ultrathin camera before image reconstruction. **f** A reconstructed image by super-resolution imaging. Photographs of **g** a commercialised compact camera (Raspberry pi camera V2) and **h** the ultrathin arrayed camera. Radial star images acquired by **i** the commercialised camera and **j** the single channel of the ultrathin arrayed camera. **k** Reconstructed image from several channels. **l** Comparison of MTF curves measured by the commercialised compact camera image, the single channel image of ultrathin arrayed camera, and the reconstructed image

A high-resolution image was finally reconstructed by using a multi-frame super-resolution algorithm (Fig. 4). The ultrathin camera offers array images of a single dice displayed on an LED panel 10 cm from the camera (Fig. 4a). The experimental results also demonstrate that the MTF for the reconstructed images logarithmically increases with the number of merged channel images (Fig. 4b, c). In other words, the MTF50 for a single channel image of an ultrathin camera is initially 129 cycles mm^−1^. However, the MTF50 for the reconstructed image merged from 15 channel images is increased by 1.57 times, i.e., 202 cycles mm^−1^ and thus exhibits a clear increase in edge sharpness. During the super-resolution imaging, the growth of the image quality is reduced by the number of merged channel images due to the logarithmic growth relationship of the computational efficiency and merged image quality^28,29^. A reconstructed image also represents a similar colour reproduction of a target object (Fig. 4e, f). The colour difference between the target and captured images was calculated by using the Euclidean distance, which exhibits a normalised colour difference of 0.31 for the single channel image and 0.08 for the reconstructed image. The reconstructed images from the ultrathin arrayed camera were further compared with those from a commercialised compact camera (Raspberry pi camera V2, 8 MP) with a comparable f-number lens (Fig. 4g–l, Supplementary Table S1). The MTF50 for the reconstructed image is 47% of that of the commercialised camera, i.e., 430 cycles mm^−1^. The relative illumination for the commercialised camera is 97% from the image centre to the corner and that of the reconstructed image is 83% (Supplementary Fig. S4). The use of the chief ray angle (CRA)-corrected CMOS ISA, matching for a commercialised lens, either reduces the resolution of the reconstructed image or enhances the colour difference for the ultrathin arrayed camera. The problem influences MTF reduction during super-resolution imaging, and the issue can be solved with the use of the CRA redesigned CMOS ISA for the iMLAs. For instance, the MTF50 for 15 reconstructed frame images acquired on the same single channel of an ultrathin camera at a constant time interval is increased by 2.01 times, i.e., 252 cycles mm^−1^, without the CRA problem (Supplementary Fig. S5). The difference in the normalised Euclidean distance between the reconstructed image and that of a commercialised camera is 0.03, and the result means a nearly perceptually uniform space. The pinhole diameter affects the minimum illumination intensity for target detection (Supplementary Fig. S6). The minimum illumination intensity for object recognition of the commercialised camera is 0.05 lux and that of the ultrathin arrayed camera is 0.1 lux due to the small pinhole diameter of the ultrathin arrayed camera. However, the ultrathin camera exhibits a substantial improvement of 5.41 times in the TTL as well and 1.5 times in the FOV compared to a commercial camera. Moreover, the image reconstruction of the super-resolution algorithm offers a considerable enhancement in colour similarity, contrast and edge sharpness.

In summary, we have successfully demonstrated biologically inspired ultrathin arrayed cameras for high-contrast and high-resolution imaging. The MOE consists of iMLAs and MAAs, which were fabricated at the wafer level by using repeated photolithographic patterning of black and transparent polymer resin. This unique configuration allows effective removal of the optical crosstalk between microlenses and substantially reduces the camera thickness down to a TTL of 740 μm. The ultrathin arrayed camera has successfully demonstrated high-contrast and high-resolution imaging by merging channel images based on the multi-frame super-resolution imaging method. Compared to commercial compact or mobile cameras, the ultrathin arrayed camera exhibits exceptional figures of merit, including image resolution, FOV, TTL and cost-effectiveness. This novel ultrathin camera module provides new opportunities for diverse mobile, surveillance, or medical applications.

## Materials and methods

### Experimental setup and performance evaluation

The fully packaged ultrathin arrayed camera was fixed to an optical mount integrated with a rail, and the movable mount with an LED display panel was installed on the rail. The FOV was measured by the distance to a grid target, *H*, and the width of the target image, *W* (Supplementary Fig. S2); FOV = *2* tan *(W/H)*. The image sensor setting was fixed at ISO 400, a shutter speed of 1/60 sec, and frame rate of 30 fps. The image data captured with the camera were transferred to the single-board processor (Raspberry pi 3 Model B + , raspberry pi), and the image was automatically constructed by embedded software. The slanted-edge method was used to evaluate the MTF of the captured images. This method was described in standard ISO 12233, and Quick MTF software was used to obtain the quantitative values.

### Image reconstruction algorithm

The array images of individual channels were cropped for image stacking. Each cropped image was registered to one reference image to seek common centre values by calculating motion vectors^30^. The registered images were reconstructed to higher-resolution images using the following the cost function using low-resolution input images *Y*:

1 $${\widehat{\underline {X}}} = \mathop {\rm{min}}\limits_{\underline {X} } \sum \limits_{k = 1}^N \left\Vert F_k {\underline {X}} - Y_{k} \right\Vert_p^p + \lambda \gamma \left({\underline {X}} \right)$$

where *p* = 2, *F*_*k*_ is the operator of the geometric motion between the high-resolution image *X* and the *k*^*th*^ low-resolution array image *Y*_*k*_, and *λ* is the regularization factor. The total variation in the image was calculated using the regularizer of bilateral-TVL1 *γ* $\left( {\underline X } \right)$:

2 $$\gamma \left( {\underline X } \right) = \mathop {\sum }\limits_{l = 0}^P \mathop {\sum }\limits_{m = 0}^P \alpha ^{m + l}\left\| {\underline X - S_x^lS_y^m\underline X } \right\|_1$$

where *α* is a scale parameter and *S* is the shift operator in the x or y direction.

## Supplementary information

Supplementary Information for Biologically Inspired Ultrathin Arrayed Camera for High-contrast and High-resolution Imaging
