# Supplementary material for: Biologically inspired ultrathin arrayed camera for high-contrast and high-resolution imaging
Source: Light Sci Appl. 2020 Feb 27;9:28. doi: 10.1038/s41377-020-0261-8 (PMC7046746; doi:10.1038/s41377-020-0261-8)
Supplement: Supplementary file 1 — Supplementary Information for Biologically Inspired Ultrathin Arrayed Camera for High-contrast and High-resolution Imaging [file 41377_2020_261_MOESM1_ESM.docx]

**Supplementary Information**

**Biologically Inspired Ultrathin Arrayed Camera for High-contrast and High-resolution Imaging**

Kisoo Kim^1,2^, Kyung-Won Jang^1,2^, Jae-Kwan Ryu^3^ and Ki-Hun Jeong^1,2^*

*^1^Department of Bio and Brain Engineering, Korea Advanced Institute of Science and Technology (KAIST), 291 Daehak-ro, Yuseong-gu, Daejeon 34141, Republic of Korea*

*^2^KAIST Institute for Health Science and Technology, KAIST, Daejeon 34141, Republic of Korea*

*^3^Unmanned/Robotic Systems Lab., LIG Nex1 Co. Ltd, Seongnam 13488, Republic of Korea*

*email: kjeong@kaist.ac.kr

**
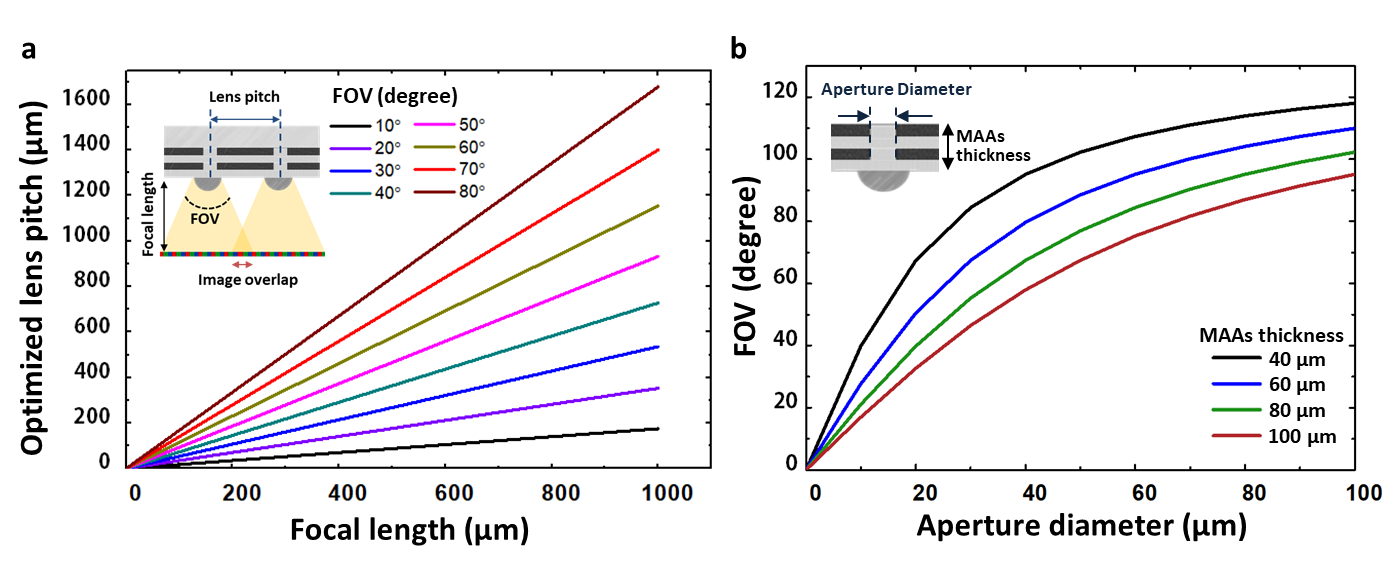
**

**Supplementary Figure S1 | Parametric design of MLA pitch and MAAs.** (a) The calculated pitch of MLA to minimize the overlap of array images on the image plane of CMOS image sensor. The optimal pitch linearly increases with both the focal length and the FOV of MLAs. In this experiment, the optimal pitch of MLA was set to 238 μm for 170 μm in focal length and 70 degrees in FOV. (b) Calculated FOV depending on the aperture diameter and the thickness of MAAs. Based on this calculation, the FOV was set to 70 degrees for the MAAs thickness of 60 μm and the aperture diameter of 35 μm for the experiment. The FOV can be monotonically and precisely controlled by increasing the thickness of MAAs.

**
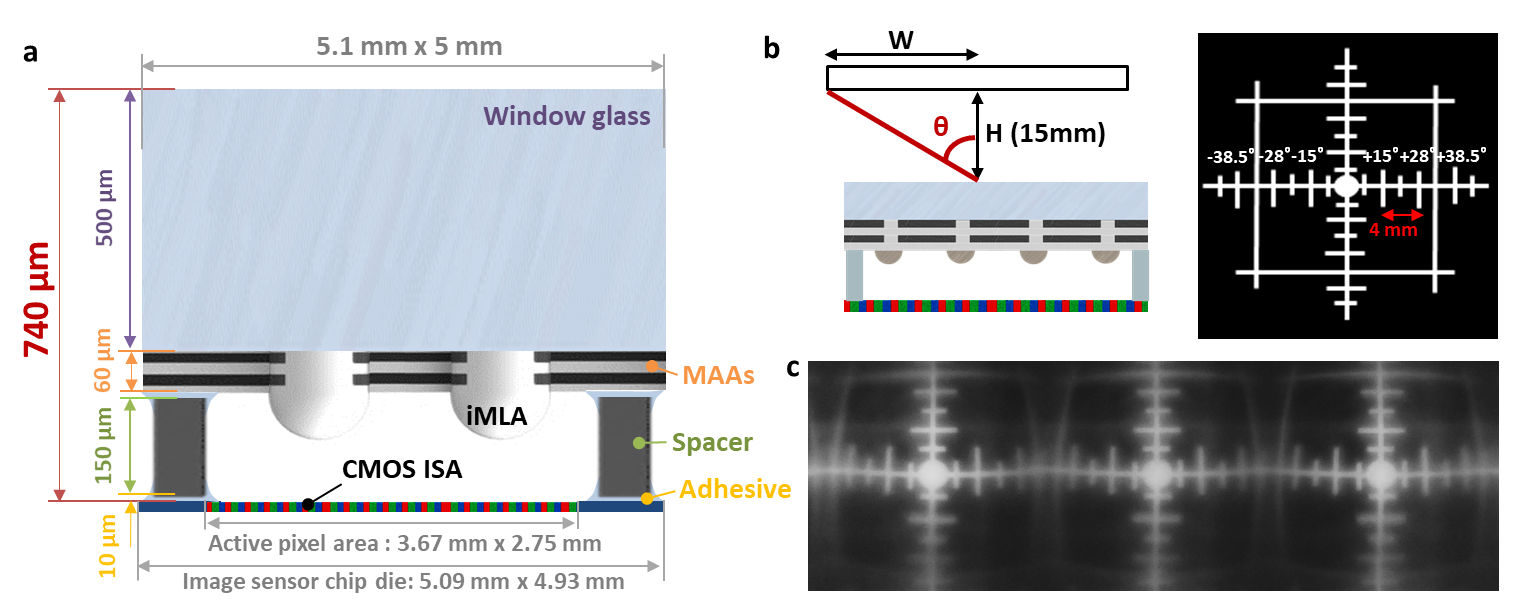
**

**Supplementary Figure S2 | Physical dimension and FOV measurement of biologically inspired ultrathin arrayed camera.** (a) The physical dimension of the ultrathin arrayed camera. The total track length is 740 μm, which includes 7~10 μm thick adhesive resin, 150 μm thick gap spacer, 60 μm thick MAAs, and 500 μm thick window glass. The total track length can be further reduced by thinning the window glass. (b) A schematic illustration of an experimental setup and a grid target for the FOV measurement. The FOV was calculated through the distance to target (H) and the imaging width (W). *FOV = 2 tan (W/H)*. (c) The grid target images captured by the ultrathin arrayed camera. The measured FOV of each lens is 73 degrees.

**
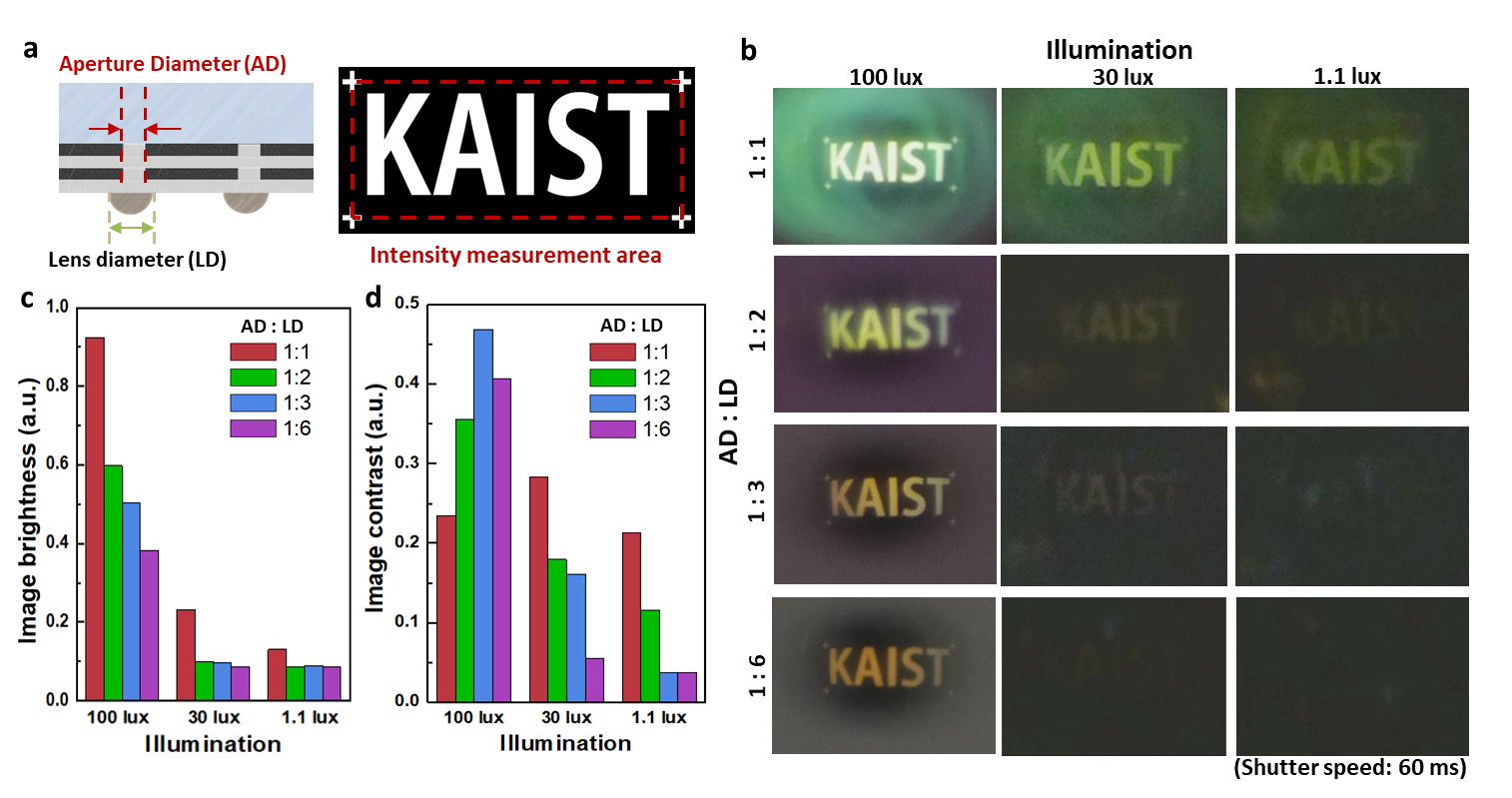
**

**Supplementary Figure S3 | Image brightness and contrast depending on the aperture diameter.** (a) A schematic illustration for the iMLAs with the MAAs and a target object. The target object was displayed on a led panel. (b) Captured images depending on the LED illumination intensity and the aperture diameter (AD), i.e., the light absorber pinhole diameters. All the images were captured at constant lens diameter (LD) of 100 μm depending on the AD, i.e., LD/AD of 1~6. The lens glare phenomenon appears at the 1:1 ratio of AD and LD due to excessive lighting. The shutter speed of image sensor was fixed at 6ms. (c) Image brightness and (d) Image contrast, i.e., *C = (L_max_-L_min_)/(L_max_+L_min_)*, depending on the LD/AD and the illumination intensity. The 1:3 ratio of AD and LD shows the maximum contrast at 100 lux illumination. The maximum contrast values in the 1:1 ratio of AD/LD were measured at low illumination such as 30 lux and 1.1 lux.

**
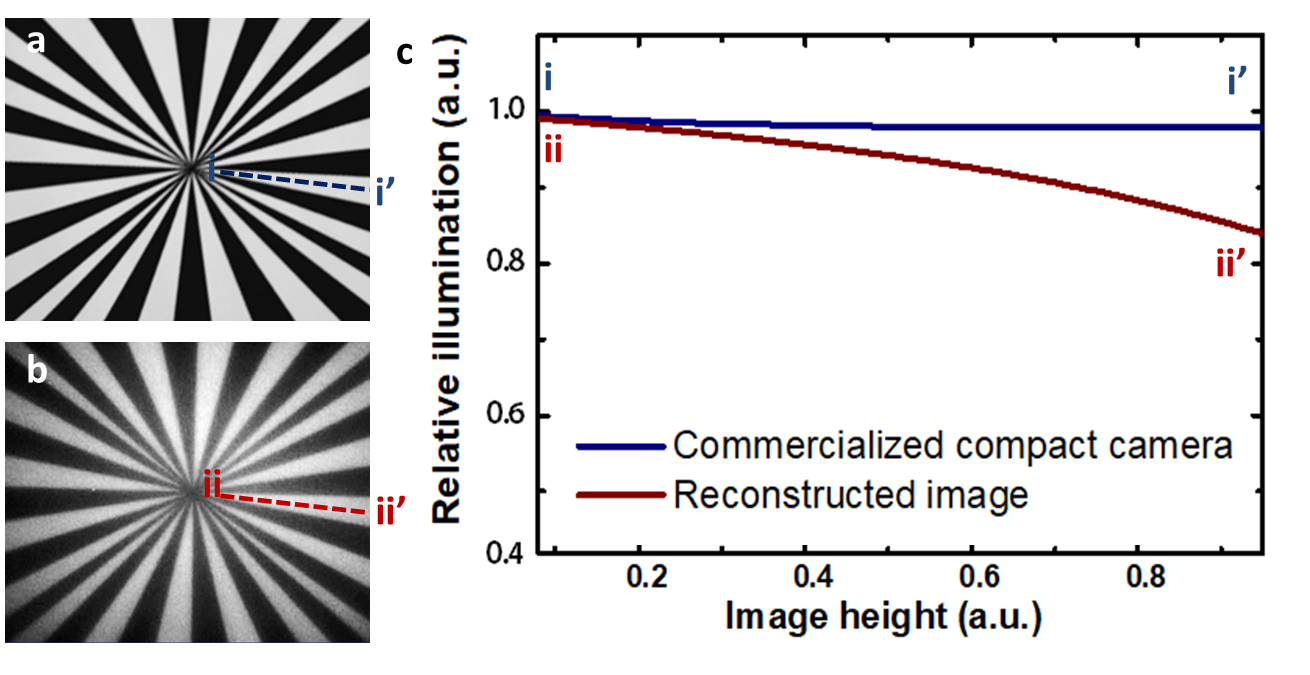

Supplementary Figure S4 | Relative illumination through the commercialized camera and the ultrathin arrayed camera.** (a) A radial star image captured through the commercialized compact camera. (b) The reconstructed image from channel images of ultrathin arrayed camera. (c) The corresponding normalized relative illumination along line i-i′, ii-ii′ to measure vignetting quantitatively.

**
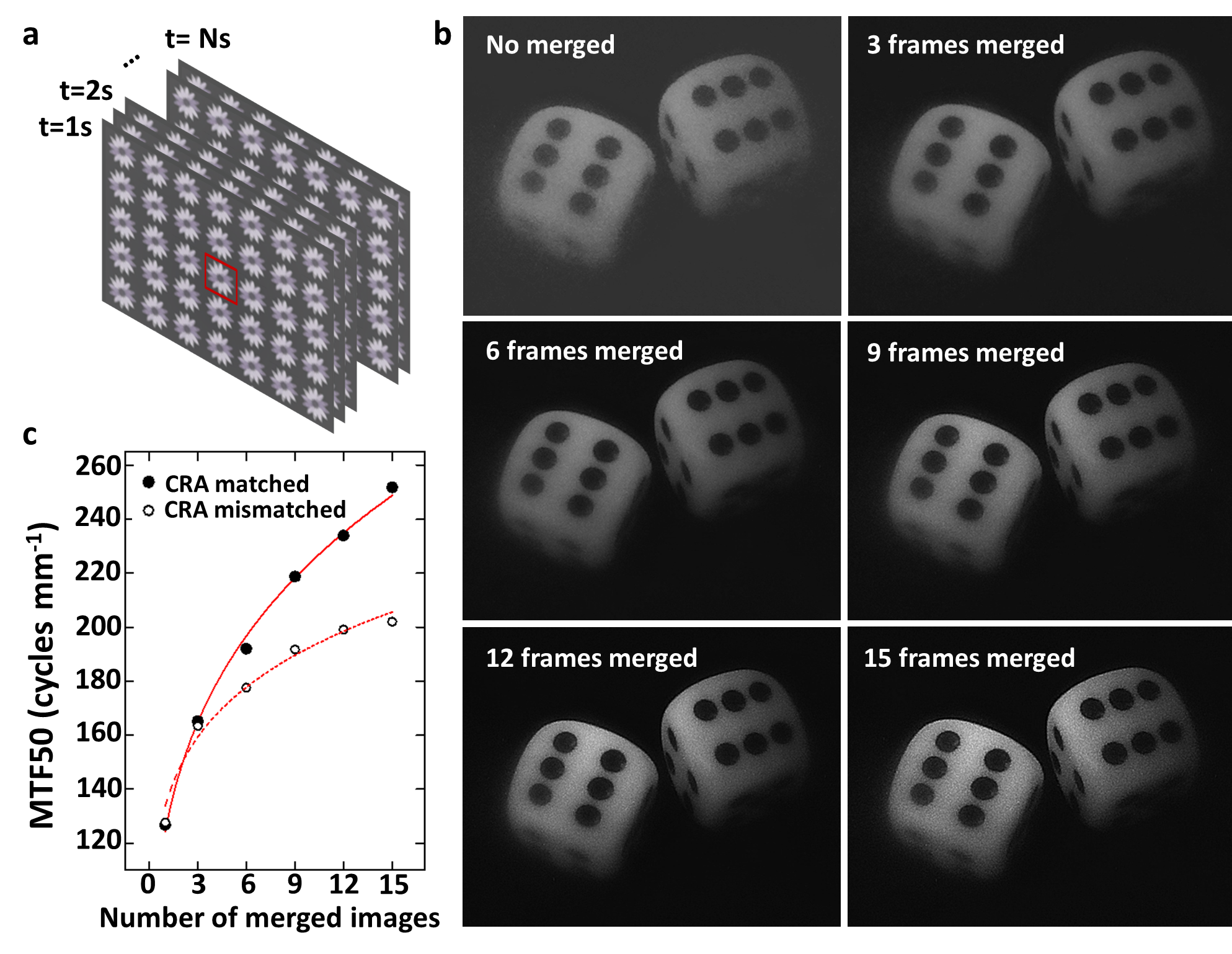
**

**Supplementary Figure S5 | Super-resolution imaging by multi-frame images.** (a) A schematic illustration of super-resolution imaging from still images acquired at a constant time interval, 1 second. (b) Reconstructed images according to the number of multi-frame images by using super-resolution imaging. (c) The graph of MTF50 for the number of integrated images. The MTF50 is enhanced than that of reconstructed image through the array images, i.e. the CRA mismatched images, and the result clearly exhibits that the CRA problem reduce the reconstructed image resolution.

**
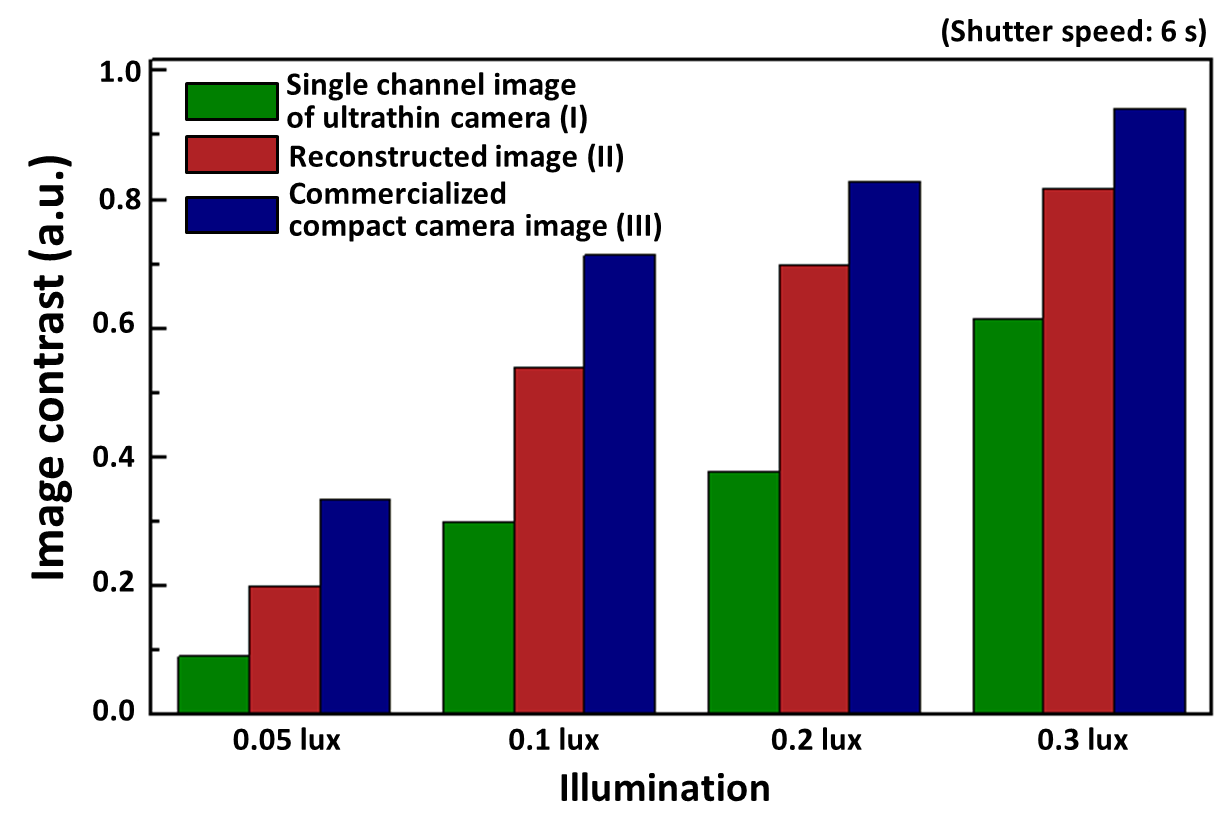
**

**Supplementary Figure S6 | Comparison of image contrast at minimum illumination intensity for target recognition.** Image contrast depending on illumination intensity. The minimum illumination intensity for target recognition is lighting intensity that captures an image with a contrast value of more than 0.3 when the shutter speed of image sensor is the maximum, 6s. The measured minimum illumination intensity of commercialized camera is 0.05 lux, and that of ultrathin camera is 0.1 lux. The image contrast for the reconstructed image is increased by 1.63 times on average.

**Supplementary Table S1 | Comparison of the specifications between the conventional compact camera and the ultrathin array camera.**

|  | **Conventional camera**  **(Raspberry pi camera V2)** | **Ultrathin arrayed camera** |
| --- | --- | --- |
| **Lens /Aperture Diameter** | 6 mm / 1.5 mm | 100 μm / 35 μm |
| **Weight** | 0.349g | 0.157g |
| **Total track length (TTL)** | 5 mm | 740 μm |
| **CMOS Image Sensor type**  **(Pixel pitch)** | Sony IMX219  (1.12 x 1.12μm) | |
| **CMOS Image Sensor sensitivity** | 186 mV∙lux^-1^·sec^-1^ | |
| **FOV** | 62.2 x 48.8 degrees | 73 x 73 degree |
| **Lens number** | Single lens | Total 35 (7 x 5) |
| **Still resolution**  **/channel** | 8 Megapixels | 45,000 Pixels |
| **Pixel resolution**  **/channel** | 3,285 x 2,464 pixels | 214 x 214 pixels |
| **Image area**  **/channel** | 3.68 x 2.76 mm | 240 x 240 μm |
| **Total Effective image area** | 10.16 mm^2^ | 1.73 mm^2^ |
| **Vignetting**  **(Relative illumination)** | 97% | 82%  (merged image) |
| **Min. illumination**  **detection intensity** | 0.05 lux  @ 6s exposure time | - 1. lux   @ 6s exposure time |
| **MTF 50** | 430 cycles∙mm^-1^ | 202 cycles∙mm^-1^  (Integrated image) |
